# Is There an Association of Vascular Disease and Atherosclerosis in Children and Adolescents With Obesity and Non-alcoholic Fatty Liver Disease?

**DOI:** 10.3389/fped.2018.00345

**Published:** 2018-11-16

**Authors:** Sara Karjoo

**Affiliations:** Department of Pediatrics, Johns Hopkins Medicine, All Children's Hospital, St. Petersburg, FL, United States

**Keywords:** pediatrics, non-alcoholic fatty liver disease, carotid intima media thickness, atherosclerosis, brachial flow mediated dilation

## Abstract

Carotid intima media thickness (cIMT) and brachial flow-mediated dilation (FMD) evaluated by ultrasound are non-invasive markers of atherosclerosis. Increased cIMT in adults has been correlated to early vascular damage. Several studies show similar correlations of elevated cIMT in children with obesity, hyperlipidemia, and metabolic syndrome. Additionally, several articles have correlated non-alcoholic fatty liver disease (NAFLD) with elevated cIMT, indicating early atherosclerosis. It is alarming that these vascular changes may be seen in children as young as 10 years of age. Children with NAFLD may also have an increased pulse wave velocity that correlates to increased arterial stiffness and increased left ventricular dimension, mass, and diastolic dysfunction. These articles are persuasive, indicating a correlation of Pediatric NAFLD and early vascular disease. However, study limitations include the use of elevated alanine aminotransferase (ALT) and echogenic changes on ultrasound that may have low accuracy to identify NAFLD. Ultrasound has low sensitivities and specificities for detection of NAFLD and therefore is not recommended for diagnosis. In comparison, studies that used liver biopsy or proton magnetic resonance spectroscopy to identify NAFLD did not find a correlation with elevated cIMT or reduction in FMD. Due to these conflicting findings, more studies looking at cIMT and FMD changes in children with NAFLD are needed with more accurate diagnostic methods for steatosis to identify if there truly is a correlation of increased liver steatosis to early atherosclerosis.

## Introduction

The prevalence of obesity in the United States has increased in the past 4 years to 1 in 5 children (1). There has also been a considerable increase in the prevalence of obesity from 2015 to 2016 in children ages 2–5 years (1). Recent data shows increased rates of obesity in children of Hispanic and African American heritage as compared to other races (1). Obesity is a disease that effects the whole body. Non-alcoholic fatty liver disease (NAFLD) is just one finding in patients with obesity. As the rates of obesity increase, so does the prevalence of NAFLD (2). NAFLD is truly a histological diagnosis defined as steatosis >5% (3), and often is clinically asymptomatic. It occurs in higher frequency in individuals of Hispanic origin (especially from South America) and of Middle Eastern origin (4, 5). It also occurs with intermediate frequency in Whites, and less commonly in Blacks (4, 5). Those individuals of Hispanic heritage with fatty liver may have a higher rate of progression to fibrosis (5). In addition, it now well-known that children with the rs738409 C>G adiponutrin/patatin-like phospholipase domain-containing 3 (PNPLA3) polymorphism gene mutation have a higher risk of severe steatosis and progression to fibrosis (6). NAFLD can progress over time to a condition called nonalcoholic steatohepatitis (NASH), which has features of ballooning steatosis, fibrosis, and inflammation (4). There are concerns that NASH in young adults will become the leading cause of liver transplants in the future (7). In addition to liver damage, could the finding of NAFLD be correlated to early cardiac disease?

Historically, Berenson et al. published their post mortem autopsy findings of individuals ages 2–39 years, showing that aortic or coronary artery fatty streaks and fibrous plaques were strongly correlated to elevated body mass index (BMI), elevated blood pressure, and mixed hyperlipidemia (8). One non-invasive method to assess vascular changes is evaluation of carotid intima media thickness (cIMT) via carotid ultrasound. Increased cIMT in adults has been correlated to early vascular damage (9, 10). In addition, there are findings of coronary artery disease and altered ventricular function in adults with NAFLD (11–13). Adults with NAFLD were found to have coronary artery stenosis, higher coronary artery calcium, and all types of plaque (calcified and non-calcified) as compared to controls (14). Similarly, the authors of a systematic review and meta-analysis study showed increased left ventricular mass in adult patients with NAFLD as compared to controls (15). Clearly, there are many well-described associations of atherosclerosis and structural heart disease in adults who have NAFLD.

As discussed previously, there are racial difference with the prevalence of NAFLD. Similarly, there may be different prevalence and onset of cardiac disease in certain racial groups. In one recent adult study, the authors found that NAFLD was highest in patients who were of Hispanic heritage and lowest in Blacks and Chinese (16). Overall, the total prevalence of abdominal aortic calcification was highest in Whites, followed by Chinese, Blacks, and Hispanics (16). However, when evaluating the participants with NAFLD, the abdominal aortic calcification was highest in Hispanics, followed by Chinese, then Blacks, and finally Whites (16). The authors concluded that NAFLD did have an increased association with abdominal aortic calcification, and may affect different racial groups differently (16).

Such studies looking at the association of NAFLD and atherosclerosis with different racial groups has not been evaluated in children. This may be a possible topic for future Pediatric NAFLD research, looking at the differences and the onset of cardiac disease in various racial groups.

However, several pediatric articles have been published demonstrating the association of cIMT or brachial flow-mediated dilation (FMD) changes in children with NAFLD (17–34). In comparison, fewer and more recent articles have been published that do not show this association (35–37). The pediatric data currently published may have limitations and may not be generalizable to all children with NAFLD, due to the different onset and progression of heart disease in different racial groups. Overall, there likely is an association when taking into account the significant amount of published studies showing a link between NAFLD and vascular disease. However, better well-designed studies are needed with more accurate methods to identify NAFLD while taking into account racial onset and prevalence of hepatic steatosis. The articles published regarding the association of NAFLD with cIMT and FMD changes are reviewed in this article.

## Methods

The articles were identified with the help of an unbiased medical librarian at Johns Hopkins All Children's Hospital. Two independent searches 6 months apart was conducted. Embase and PubMed were used to identify articles that fit the criteria. The search was limited to ages 0–18 years, and key words of NAFLD, cIMT, FMD, hyperlipidemia, obesity, and atherosclerosis. Each term was used in combination to limit articles and each item was also searched independently. Articles that were relevant to this review were used. Articles were excluded if they did not have research subjects with NAFLD.

### Studies supporting association of NAFLD with elevated cIMT and low FMD

Overall, there have been more articles in the literature in support of early vascular changes like elevated cIMT and low FMD in children with NAFLD and obesity (17–34). What is interesting is that these articles used either abdominal ultrasound alone or abdominal ultrasound and elevated liver enzymes (alanine aminotransferase in most studies >40 U/L) to identify steatosis and NAFLD (17–34, 38). One study also used magnetic resonance imaging (MRI) findings as well to identify patients with steatosis (19). In some papers, a grading system was used to identify the level of steatosis or changes on liver ultrasound (18, 21–23, 26, 28, 29, 33). Many of the papers either used BMI percentile (14, 17–19, 21–23, 25, 26, 28, 32, 38, 39) or a fixed BMI >28–30 kg/m^2^ to define obesity (27, 29–31, 33). Echocardiography and carotid ultrasound were used to identify cIMT. FMD was evaluated via Doppler ultrasound imaging before and after an ischemic event caused by reduced blood flow from an inflated sphygmomanometer (19, 21, 37). All the papers excluded patients if they had systemic diseases or significant alcohol use.

There has been a series of articles by Pacifico et al. that have dominated the research in this area. In one of their studies, the authors found reduced FMD in obese children with NAFLD when exposed to ischemia (30). Children with NAFLD and obesity had elevated cIMT which was even higher if they had metabolic syndrome (30). In another study by the same group, the patients who had hepatic steatosis had higher mean and maximum cIMT measurements (23). They also found elevated cIMT was associated with elevated blood pressure, insulin resistance, NAFLD, and high triglyceride to high density lipoprotein cholesterol (TG/ HDL-c) ratio (12). The authors also demonstrated that after 12 month intervention of diet and exercise in children with NAFLD and obesity, the FMD improved but elevated cIMT did not regress (19). Failure of improvement of cIMT with lifestyle intervention is concerning, and further studies need to be done to see if this is a reproducible finding in children.

Other authors have also found similar correlations of elevated cIMT with NAFLD (18, 20–22, 25–28, 31, 33, 34), and have linked increased severity of steatosis to even higher cIMT values (18, 25), and lower FMD values (21). The inclusion of hepatic steatosis on ultrasound may improve the cIMT predictability of cardiac disease as compared to metabolic syndrome alone (28). A correlation of higher cIMT measurements was found with elevated aspartate aminotransferase to platelet ratio index (APRI), which is a marker for hepatic fibrosis (33).

Structural heart changes have also been reported such as increased thickening of the left ventricle, higher interventricular septal thickness in systole, increased left ventricular posterior wall thickness in diastole, increased left atrial and aortic diameters, higher left ventricular mass, and higher left ventricular mass index in children with NAFLD and obesity (22, 30, 31). The authors of one study looked at applanation tonometry to measure arterial stiffness via pulse wave velocity (29). They found that those patients who had NAFLD with other high risk metabolic abnormalities had greater pulse wave velocity as compared to those without metabolic complications (29).

In summary, there are many articles showing an association of early signs of structural heart disease and atherosclerosis in children with NAFLD. Perhaps practitioners need to be more concerned with the finding of NAFLD, as this may be a sign of early heart disease in children.

### Studies lacking association of NAFLD with elevated cIMT and low FMD

What is interesting is that some newer articles do not support the association of elevated cIMT and low FMD in children who have NAFLD. What is unique about these articles is the use of either magnetic resonance spectroscopy or liver biopsy to define steatosis (35–37). When comparing degree of steatosis, inflammation, and fibrosis, there was no correlation with elevated right or left cIMT values (36). Steatosis and serum ALT was not correlated to elevated cIMT or arterial wall stiffness (35) either. The only predictor was BMI for abnormalities on cIMT (36). In addition, there was no association of hepatic fat fraction and FMD changes (37).

### Diagnosis of liver steatosis, fibrosis, and NAFLD

At this time, the North American Society of Pediatric Gastroenterology Hepatology and Nutrition (NASPGHAN) clinical practice guideline for NAFLD in children recommends the use of serum ALT at the age of 10 years (40) for screening. Ultrasound has fallen out of favor for the screening and diagnosis of NAFLD due to the low sensitivities and specificities (40, 41). There are new radiologic techniques like magnetic resonance elastography with better sensitivities and specificities that are validated for the evaluation of hepatic steatosis and fibrosis (40, 42) in children. Magnetic resonance elastography also has ~88% sensitivity and 85% specificity for detecting fibrosis (43). Ultimately, the gold standard for the evaluation of liver steatosis and fibrosis is histology of liver biopsy samples (3, 38, 44). Liver biopsy or magnetic resonance imaging are more accurate but also more expensive techniques for diagnosis (43). Liver biopsy is also invasive and can have complications of bleeding and damage to the gallbladder. There is also an added cost of anesthesia and 12–24 h hospital admission for observation post liver biopsy. To reduce the cost of diagnosis, non-invasive fibrosis scores, or serum biomarkers are under evaluation. In children, thus far, fibrosis scores that are typically used in adults are not accurate in predicting liver fibrosis (43). Equally, serum fibrosis biomarkers like caspase-cleaved cytokeratin 18 (CK18) show promise in recent research studies, but need further validation before it is recommended as part of clinical practice (43).

## Conclusions

Overall, there are many studies correlating elevated BMI, hepatic steatosis, and metabolic syndrome with increased cIMT, lower FMD, possible increased arterial stiffness, and ventricular dysfunction in children. These articles predominantly used liver ultrasound with or without serum ALT to identify NAFLD. Some authors also used ultrasound grading of the echogenicity to correlate with severity of hepatic steatosis. Recently, ultrasound has fallen out of favor for screening for NAFLD due to the sensitivities and specificities of the test, and may not be an accurate way to identify hepatic steatosis. In comparison, the articles that define NAFLD with liver biopsy or proton magnetic resonance spectroscopy did not find this correlation to structural heart or vascular changes.

There is also a different prevalence of NAFLD in different racial groups. Perhaps the onset of cardiac disease, therefore, is different in different ethnic groups as well. One limitation of these studies is generalizability of the findings to all races and populations. Robust studies are needed which use more accurate diagnostic techniques for NAFLD, and take into consideration the differences of the disease frequency in different ethnic groups.

With the global health and economic impact of increased rates of obesity and therefore NAFLD in children, it is important for future research to identify if there is a correlation of hepatic steatosis to early atherosclerosis and at what age this occurs. The information from the future research would help create clinical programs for early diagnosis and intervention before significant vascular disease begins.

## Author contributions

The author confirms being the sole contributor of this work and has approved it for publication.

### Conflict of interest statement

The author declares that the research was conducted in the absence of any commercial or financial relationships that could be construed as a potential conflict of interest.
